# Subcellular localization of fibroblast growth factor receptor type 2 and correlation with *CTNNB1* genotype in adrenocortical carcinoma

**DOI:** 10.1186/s13104-020-05110-5

**Published:** 2020-06-10

**Authors:** Matthias Haase, Anne Thiel, Ute I. Scholl, Hany Ashmawy, Matthias Schott, Margret Ehlers

**Affiliations:** 1 Division for Specific Endocrinology, Medical Faculty, University Hospital Duesseldorf, Moorenstr 5, 40225 Düsseldorf, Germany; 2 Department of Nephrology, Medical Faculty, University Hospital Duesseldorf, 40225 Düsseldorf, Germany; 3 Charité - Universitätsmedizin Berlin, corporate member of Freie Universität Berlin, Humboldt-Universität zu Berlin, and Berlin Institute of Health, Department of Nephrology and Medical Intensive Care and BIH Center for Regenerative Therapies, Augustenburger Platz 1, 13353 Berlin, Germany; 4 Berlin Institute of Health (BIH), Anna-Louisa-Karsch-Str. 2, 10178 Berlin, Germany; 5 Department of Surgery (A), Medical Faculty, University Hospital Duesseldorf, 40225 Düsseldorf, Germany

**Keywords:** Adrenocortical carcinoma, FGFR2, Beta catenin, *CTNNB1*

## Abstract

**Objective:**

Fibroblast growth factor receptor (FGFR) 2 regulates the development of the adrenal gland in mice. In addition, FGFR2-mediated signalling has been shown to prevent apoptosis and to enhance proliferation in adrenocortical precursor cells. The activation of the Wingless/Int-1 (WNT)/beta catenin pathway as a key mechanism of adrenocortical tumourigenesis has been linked to FGFR2 signalling in other cell types. Therefore we hypothesised that FGFR2 expression may also play a role in adrenocortical carcinoma (ACC). We conducted a pilot study and analysed protein expression of FGFR2 in 26 ACCs using immunohistochemistry technique. Data on the *CTNNB1* mutation status and clinical data were correlated to the expression of FGFR2.

**Results:**

We observed a high variability in FGFR2 expression between the different tumour samples. There was a subset of ACC with comparatively high nuclear expression of FGFR2. We did not find a clear association between the *CTNNB1* mutational status or clinical features and the FGFR2 expression. We conclude that FGFR signalling plays a role in adrenocortical carcinoma. Our data encourages further investigations of FGFR signalling in ACC, especially since new inhibitors of FGFR signalling are already entering clinical trials for the treatment of other cancer types.

## Introduction

Adrenocortical carcinoma is a rare malignant tumour with a poor prognosis. To date, complete resection is the only curative therapeutic option. Unfortunately, adrenocortical carcinoma is frequently diagnosed in an advanced stage. Established therapeutic measures for these patients include adrenolytic therapy with mitotane and a combination chemotherapy consisting of cisplatin, etoposide and doxorubicin. However, these therapies often allow for tumour control only for a limited period, reflected by low 5-year survival in advanced tumour stages [1]. Therefore, there is need for the investigation of new therapeutic approaches. Different fibroblast growth factors have been demonstrated to increase proliferation and growth in adrenocortical cells [2–4]. The transcriptional coactivator CITED2 (Cbp/p300-interacting transactivator 2), which is important for the development of the adrenal glands, is under control of basic fibroblast growth factor in adrenocortical cells [5].

Fibroblast growth factor receptor type (FGFR) 2, coded on human chromosome 10q26, is a tyrosine kinase receptor composed of an extracellular immunoglobulin-like domain and an intracellular tyrosine kinase domain [6, 7].

FGFR2 has also been implicated in the development of the adrenal cortex in mice. Lack of receptor subtypes causes impairment of adrenal growth and differentiation during adrenal development [8]. FGFR2 signalling increases proliferation and acts as a negative regulator of apoptosis in adrenocortical precursor cells [9]. Recent studies also identified aberrant FGFR2 signalling as an important factor in oncogenesis and as a potential drug target in different cancer types [10]. Activation of the WNT/beta catenin pathway is regarded as key mechanism in the pathogenesis of ACC. About 10 to 15% of ACCs carry activating mutations of *CTNNB1* that cause abnormal accumulation of beta catenin [11]. Interestingly, in other cell types, FGFR signalling has been demonstrated to activate canonical WNT signalling by phosphorylation of beta catenin and by increasing the cellular response to WNT [12]. In this context, we asked whether FGFR2-expression is also present in adrenocortical carcinomas and if it is associated with the mutational status of *CTNNB1* and clinical characteristics.

## Main text

### Materials and methods

#### Patients and tumour samples

We screened our local database at the University Hospital Duesseldorf and identified n = 26 patients since 1990 with adrenocortical carcinoma and available paraffin-embedded tumour samples for immunohistological studies. Clinical data on sex, age, survival (in part right censored) and hormonal activity was collected if available. Definition of hormonal activity (cortisol and androgen excess) was based on clinical features and laboratory results including clinical signs of hypercortisolism or hyperandrogenemia, elevated serum androgens, pathological dexamethasone suppression test and analysis of free urinary cortisol. Due to the retrospective design of the study, complete data was not available in all cases.

#### Reverse transcription polymerase chain reaction (RT-PCR)

Ribonucleic acids (RNAs) of normal adult human adrenal cortices were obtained from BD Biosciences Clontech (Palo Alto, CA, USA; one vial). Total RNAs from NCI-H295R cells (n = 3) were extracted using the RNeasy Plus Mini Kit (Qiagen, Hilden, Germany). RNAs were reversely transcribed with the High-Capacity cDNA (complementary deoxyribonucleic acid) Reverse Transcription Kit (Applied Biosystems) according to the manufacturer’s instructions (note: one mixed RNA of human adrenal cortices was used for three preparations of cDNA). RT-PCR was performed using the Step One Plus System (Applied Biosystems). The 20 µl PCR included cDNA template (500 ng) diluted in RNase-free water (final volume: 9 µl), 20× TaqMan Gene Expression Assay (1 µl) and 2× TaqMan Gene Expression Universal Master Mix (10 µl, Applied Biosystems). The reactions were incubated in a 96-well optical plate at 95 °C for 10 min, followed by 40 cycles of 95 °C for 15 s and 60° for 1 min. RT-PCR was performed three times with all templates in triplicate, and average cycling threshold (CT) units were obtained as the average of the results. The threshold cycle (CT) is defined as the cycle number at which the fluorescence passes the fixed threshold.

#### Immunohistochemistry

Paraffin embedded tissue specimens of adrenocortical carcinoma were examined by immunohistochemistry technique using an affinity purified polyclonal antibody raised against the c-terminal cytoplasmatic domain of the FGFR2. Specificity of the antibody has been described before [13].

Tissue sections were deparaffinised and rehydrated using Xylene and a descending alcohol series followed by antigen retrieval using target retrieval solution (Dako). For staining, the two-step immunohistochemistry staining technique Dako EnVision^®^ + System-HRP (AEC) was used. According to the manufacturer’s instructions, endogenous peroxidase activity was blocked for 5 min. Subsequently, sections were incubated with primary polyclonal FGFR2 antibody raised in rabbit [alternative name: Bek (C-17); 1:100 diluted; Santa Cruz Biotechnology] at room temperature for 30 min. As a negative control, the primary antibody was omitted. Thereafter, the sections were incubated for 30 min at room temperature with horseradish peroxidase (HRP)-labeled polymer conjugated secondary antibody against rabbit Immunoglobulin G (IgG, Dako). For signal detection, the sections were incubated with the ready-to-use aminoethyl carbazole (AEC) substrate-chromogen solution for 30 min according to the manufacturer’s protocol [Dako EnVision^®^ + System-HRP (AEC)] and then washed with distilled water. Sections were counterstained with hematoxylin (Merck) for 1 min, followed by washing with distilled water. Finally, specimens were mounted and coverslipped with Faramount (Dako). Photomicrographs were taken with an AxioCam MRc camera (Zeiss) using a microscope (Axioskop, Zeiss).

#### Analysis of *CTNNB1* mutation status

Characteristics of seven patients (patients 17, 19–23, 25) from this study, including their mutational status, were published before [14]. Tumour and corresponding normal DNA was extracted from formalin-fixed paraffin-embedded tissue (FFPE) and underwent exome sequencing on the Illumina platform. In 17 cases (patients 1–16, 18), tumour DNA was isolated from FFPE cores using the BiOstic FFPE Tissue DNA Isolation Kit (MO BIO Laboratories, Carlsbad, CA, USA), repaired with the PreCR Repair Mix (New England Biolabs, Beverly, MA, USA), and purified with the Agencourt Ampure XP PCR Purification System (Agencourt Bioscience, Beverly, MA, USA) according to the manufacturers’ instructions. Routine PCR amplification of the *CTNNB1* mutation hot spot (exon 3) was performed using specific primers. In cases where larger template regions failed to amplify with *CTNNB1*_F (5′-GCTGATTTGATGGAGTTGGAC-3′) and *CTNNB1*_R (5′-CAGGACTTGGGAGGTATCCA-3′) (AA6-63), shorter template regions were amplified with *CTNNB1*E3shortf (5′-GAACCAGACAGAAAAGCGGC-3′) and *CTNNB1*E3shortr (5′-CCTCAGGATTGCCTTTACCACT-3′) (AA15-53). PCR purification and direct bidirectional Sanger sequencing were performed (Beckman Coulter Genomics, UK). All identified mutations were confirmed in independent PCRs. In sample 2, the generation of an amplicon failed for any of the primer pairs tested. In two further cases (patients 24, 26), results of mutation analysis were not available.

#### Statistical analyses

Prism software (PRISM 6, GraphPad Software, Inc., La Jolla, CA, USA) was used for calculation of statistical significances: mRNA expression data was not normally distributed and therefore analyzed using the Kruskal–Wallis test and Dunn’s multiple comparison test. *P*-values < 0.05 were considered as significant.

### Results

We analysed tumour specimens of 14 female and 12 male patients. Mean age of patients at diagnosis was 51 ± 15 years. Data of hormonal activity was available only in 9 out of 26 (34.6%) patients (cortisol n = 4, cortisol and androgen n = 4, androgen n = 1). Survival data was available in 15 out of 26 (57.7%) patients and was right censored in 11 out of 15 patients (73.3%). Median survival was 30 months. Data on *CTNNB1* mutation status was available in 23 out of 26 patients (88.5%). 6 out of 23 patients (26.1%) harboured an activating *CTNNB1* mutation. Baseline data are summarized in Table 1.

**Table 1:** Clinical data and mutation status of *CTNNB1* in correlation with the expression pattern of FGFR2 in adrenocortical carcinoma samples (n = 26)

Sample number	Sex [F/M]	Age [range in years]	Survival [months]	Ki-67 [%]	Hormonal activity	Nuclear staining	Cytoplasmatic staining	*CTNNB1*-mutation
1	F	30–40				+	+	No mutation
2	F	40–50	> 132		Cortisol	0	+	No PCR-amplicon
3	F	20–30	> 9			0	+	No mutation
4	M	40–50				+	+	p.S45P
5	M	80–90				0	+	No mutation
6	F	40–50				0	+	No mutation
7	M	60–70				0	+	No mutation
8	M	50–60	> 48	30–40	Cortisol, androgen	+	+	No mutation
9	M	30–40	48		Cortisol, androgen	+	+	No mutation
10	F	40–50	> 360			+	+	No mutation
11	M	20–30	> 30			+	+	No mutation
12	F	30–40	> 22		Androgen	+	+	p.S33P
13	F	40–50				+	+	No mutation
14	M	60–70				+	+	No mutation
15	M	40–50				+	+	p.T41A
16	F	50–60	99			+	+	p.A5V
17	F	30–40	> 151	60		+	+	No mutation^a^
18	M	60–70				0	+	No mutation
19	F	60–70	25			+	+	No mutation
20	F	40–50	> 89	5–10	Cortisol, androgen	0	+	No mutation
21	M	50–60	> 4		Cortisol	+	+	No mutation
22	F	70–80	> 4		Cortisol	0	+	p.T41A
23	F	50–60	5		Cortisol	0	0	p.L513F
24	F	50–60	> 6	25	Cortisol, androgen	0	0	
25	M	50–60		30–40		0	+	No mutation
26	M	70–80				+	+	

^a^This tumour specimen harbours a TP53 Cy135Phe mutation; blank field: data not available; survival: > indicates right censored data. Data on age is presented as a range to protect patient’s anonymity

**Fig. 1:**
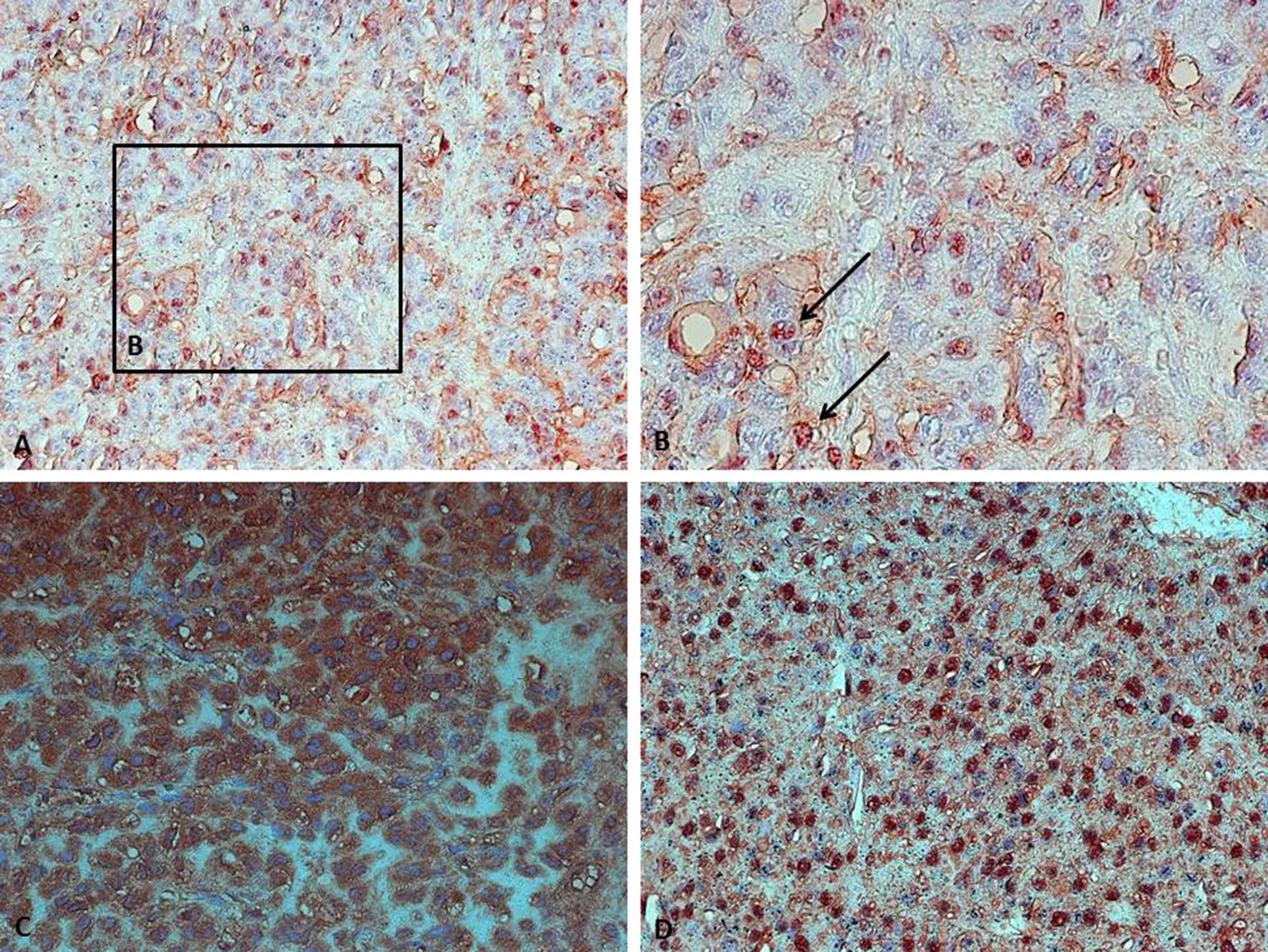
We observed different staining patterns in adrenocortical carcinomas. **a**, **b** Dot-Like expression of FGFR2 in the nuclei. An association with chromatin seems possible. **c** Strong cytoplasmic expression while nuclei are not stained. **d** Strong nuclear expression in a subset of cells and weak nuclear expression in another subset of cells. Images were captured using ×100 magnification

Most of the adrenocortical carcinomas showed cytoplasmatic (24 out of 26; 92.3%) and/or nuclear staining (15 out of 26; 57.7%) of FGFR2 (see overview in Additional file 1). Nuclear expression of FGFR2 was observed in both female and male tumour specimens. No FGFR2 immunoreactivity was detectable in two tumours (patient 23 and 24). There was no clear association of the mutational status of the *CTNNB1* gene and FGFR2 immunoreactivity. Results of immunohistochemistry are demonstrated in Fig. 1. One tumour sample (patient 17) with high cytoplasmatic and nuclear expression of FGFR2 harboured a tumour protein 53 (TP53) mutation (Cys135Phe). One sample (patient 12) included also normal adrenal tissue. The adjacent tumour tissue showed higher expression of FGFR2 as compared with the normal adrenal cortex. Using RT-PCR we could demonstrate messenger ribonucleic acid (mRNA) expression of FGFR2 in both normal adrenal cortex and in the human adrenocortical cell line NCI-H295R (see data in Additional file 2).

### Discussion

The study was designed as a pilot study to identify FGFR2 as a potential target for further investigations in adrenocortical carcinoma. To our knowledge, this is the first study of FGFR2 protein expression and localization in human adrenocortical carcinoma species. The data support the hypothesis that FGFR signalling is relevant for adrenocortical cancer, as it has already been demonstrated for other tumour entities before. The most striking result was the difference in nuclear expression of FGFR2 between the different tumour samples. While part of the tumour samples did not show nuclear expression, there was a subset of tumours with high nuclear FGFR2 expression.

The finding of nuclear expression of FGFR2 has been observed before in other tumour entities such as breast cancer [15]. The role of nuclear localisation has not yet been understood. It has been discussed that nuclear localisation of FGFR2 in breast cancer cells may be associated with a more differentiated phenotype [15]. During embryonic development of mice, fibroblast growth factor (FGF) 9 induced nuclear expression of FGFR2 in XY gonads but not in XX gonads and was associated with increased proliferation [13]. However, in our study nuclear localization of FGFR2 was observed in both male and female tumour species.

The activation of the WNT/beta catenin pathway represents a key mechanism in adrenocortical tumourigenesis [14, 16]. Interestingly, FGFR-signalling has been linked to the activation of WNT/beta catenin pathway [12]. Therefore it can be speculated whether FGFR-signalling contributes to adrenocortical tumour growth by the upstream activation of WNT/beta catenin independently from activating mutations of *CTNNB1*.

However, we did not find a clear association of the mutational status of *CTNNB1* or distinct clinical features with FGFR2 expression, although most of the tumours with the highest nuclear FGFR2 expression did not harbour a *CTNNB1* mutation. Based on differential splicing of the immunoglobulin-like domain of the FGFR2 there are two different isoforms including the epithelial variant FGFR2b and the mesenchymal variant FGFR2c. Alternative splicing and expression of FGFR2 isoforms may contribute to cancer growth in the context of epithelial to mesenchymal transition [17]. Future studies should therefore also address differences in isoform expression in ACC. In summary, the results of our pilot study encourage further investigations of FGFR-signalling in adrenocortical carcinoma. We conclude that simple quantitative measurement and comparison of expression alone is not sufficient to assess the relevance of FGFR2 signalling in ACC, since subcellular localization of FGFR2 obviously seems to play a particular role.

## Limitations

Due to the small sample size of n = 26 and limited available clinical data of the patients, we cannot exclude that existent associations are not revealed by our study. To further evaluate the association between FGFR and CTNNB1 the analysis of downstream effects of FGFR on the activation of canonical WNT-signalling would be of importance. Our study does not allow a precise quantitative analysis of FGFR2 expression in adrenocortical carcinomas but provides a qualitative first insight. Moreover, this study is based on the analysis of immunohistochemical techniques that is observer dependent. Measurement of mRNA expression was not done quantitatively based on housekeeping genes. Additional examinations including RNA-in situ hybridization would be necessary to confirm the specificity of the results.

## Supplementary information

**Additional file 1.** Overview on the expression of FGFR2 in different adrenocortical carcinomas (n = 26). Images were captured using 100× magnification.

**Additional file 2.** mRNA expression of FGFRs in the human adrenocortical cell line NCI-H295R and in normal human adrenal cortex. Asterisks indicate significant differences of the cycle thresholds (*P < 0.05, **P < 0.01). Due to the lack of references genes a valid quantitative analysis based on this data is not possible.

## Data Availability

The dataset(s) supporting the conclusions of this article is(are) included within the article (and its additional file(s)).

## Abbreviations

ACC: Adrenocortical carcinoma
AEC: Aminoethyl carbazole
cDNA: Complementary deoxyribonucleic acid
CITED2: Cbp/p300-interacting transactivator 2
CT: Threshold cycle
CTNNB1: Catenin beta 1
DNA: Deoxyribonucleic acid
FFPE: Formalin-fixed paraffin-embedded tissue
FGF: Fibroblast growth factor
FGFR: Fibroblast growth factor receptor
HRP: Horseradish peroxidase
IgG: Immunoglobulin G
mRNA: Messenger ribonucleic acid
PCR: Polymerase chain reaction
RNA: Ribonucleic acid
TP53: Tumour protein 53
WNT: Wingless/int-1

## References

[1] Libé R (2015). Adrenocortical carcinoma (ACC): diagnosis, prognosis, and treatment. Front Cell Dev Biol.

[2] Basile DP, Holzwarth MA (1993). Basic fibroblast growth factor may mediate proliferation in the compensatory adrenal growth response. Am J Physiol.

[3] Boulle N, Gicquel C, Logié A, Christol R, Feige JJ, Le Bouc Y (2000). Fibroblast growth factor-2 inhibits the maturation of pro-insulin-like growth factor-II(Pro-IGF-II) and the expression of insulin-like growth factor binding protein-2(IGFBP-2) in the human adrenocortical tumor cell line NCI-H295R. Endocrinology.

[4] Feige JJ, Vilgrain I, Brand C, Bailly S, Souchelnitskiy S (1998). Fine tuning of adrenocortical functions by locally produced growth factors. J Endocrinol.

[5] Haase M, Schott M, Bornstein SR, Malendowicz LK, Scherbaum WA, Willenberg HS (2007). CITED2 is expressed in human adrenocortical cells and regulated by basic fibroblast growth factor. J Endocrinol.

[6] Dienstmann R, Rodon J, Prat A, Perez-Garcia J, Adamo B, Felip E, Cortes J, Iafrate AJ, Nuciforo P, Tabernero J (2014). Genomic aberrations in the FGFR pathway: opportunities for targeted therapies in solid tumors. Ann Oncol.

[7] Katoh Y, Katoh M (2009). FGFR2-related pathogenesis and FGFR2-targeted therapeutics. Int J Mol Med.

[8] Guasti L, Candy Sze WC, McKay T, Grose R, King PJ (2013). FGF signalling through Fgfr2 isoform IIIb regulates adrenal cortex development. Mol Cell Endocrinol.

[9] Häfner R, Bohnenpoll T, Rudat C, Schultheiss TM, Kispert A (2015). Fgfr2 is required for the expansion of the early adrenocortical primordium. Mol Cell Endocrinol.

[10] Chae YK, Ranganath K, Hammerman PS, Vaklavas C, Mohindra N, Kalyan A, Matsangou M, Costa R, Carneiro B, Villaflor VM, Cristofanilli M, Giles FJ (2017). Inhibition of the fibroblast growth factor receptor (FGFR) pathway: the current landscape and barriers to clinical application. Oncotarget.

[11] Maharjan R, Backman S, Åkerström T, Hellman P, Björklund P (2018). Comprehensive analysis of *CTNNB*1 in adrenocortical carcinomas: identification of novel mutations and correlation to survival. Sci Rep.

[12] Krejci P, Aklian A, Kaucka M, Sevcikova E, Prochazkova J, Masek JK, Mikolka P, Pospisilova T, Spoustova T, Weis M, Paznekas WA, Wolf JH, Gutkind JS, Wilcox WR, Kozubik A, Jabs EW, Bryja V, Salazar L, Vesela I, Balek L (2012). Receptor tyrosine kinases activate canonical WNT/β-catenin signaling via MAP kinase/LRP6 pathway and direct β-catenin phosphorylation. PLoS ONE.

[13] Schmahl J, Kim Y, Colvin JS, Ornitz DM, Capel B (2004). Fgf9 induces proliferation and nuclear localization of FGFR2 in Sertoli precursors during male sex determination. Development.

[14] Juhlin CC, Goh G, Healy JM, Fonseca AL, Scholl UI, Stenman A, Kunstman JW, Brown TC, Overton JD, Mane SM, Nelson-Williams C, Bäckdahl M, Suttorp AC, Haase M, Choi M, Schlessinger J, Rimm DL, Höög A, Prasad ML, Korah R, Larsson C, Lifton RP, Carling T (2015). Whole-exome sequencing characterizes the landscape of somatic mutations and copy number alterations in adrenocortical carcinoma. J Clin Endocrinol Metab.

[15] Martin AJ, Grant A, Ashfield AM, Palmer CN, Baker L, Quinlan PR, Purdie CA, Thompson AM, Jordan LB, Berg JN (2011). FGFR2 protein expression in breast cancer: nuclear localisation and correlation with patient genotype. BMC Res Notes.

[16] Tissier F, Cavard C, Groussin L, Perlemoine K, Fumey G, Hagneré AM, René-Corail F, Jullian E, Gicquel C, Bertagna X, Vacher-Lavenu MC, Perret C, Bertherat J (2005). Mutations of beta-catenin in adrenocortical tumors: activation of the Wnt signaling pathway is a frequent event in both benign and malignant adrenocortical tumors. Cancer Res.

[17] Ranieri D, Rosato B, Nanni M, Magenta A, Belleudi F, Torrisi MR (2016). Expression of the FGFR2 mesenchymal splicing variant in epithelial cells drives epithelial-mesenchymal transition. Oncotarget.

