# Supplementary figures and images for: Subcellular localization of fibroblast growth factor receptor type 2 and correlation with CTNNB1 genotype in adrenocortical carcinoma
Source: BMC Res Notes. 2020 Jun 10;13:282. doi: 10.1186/s13104-020-05110-5 (PMC7288682; doi:10.1186/s13104-020-05110-5)

## Slide 1
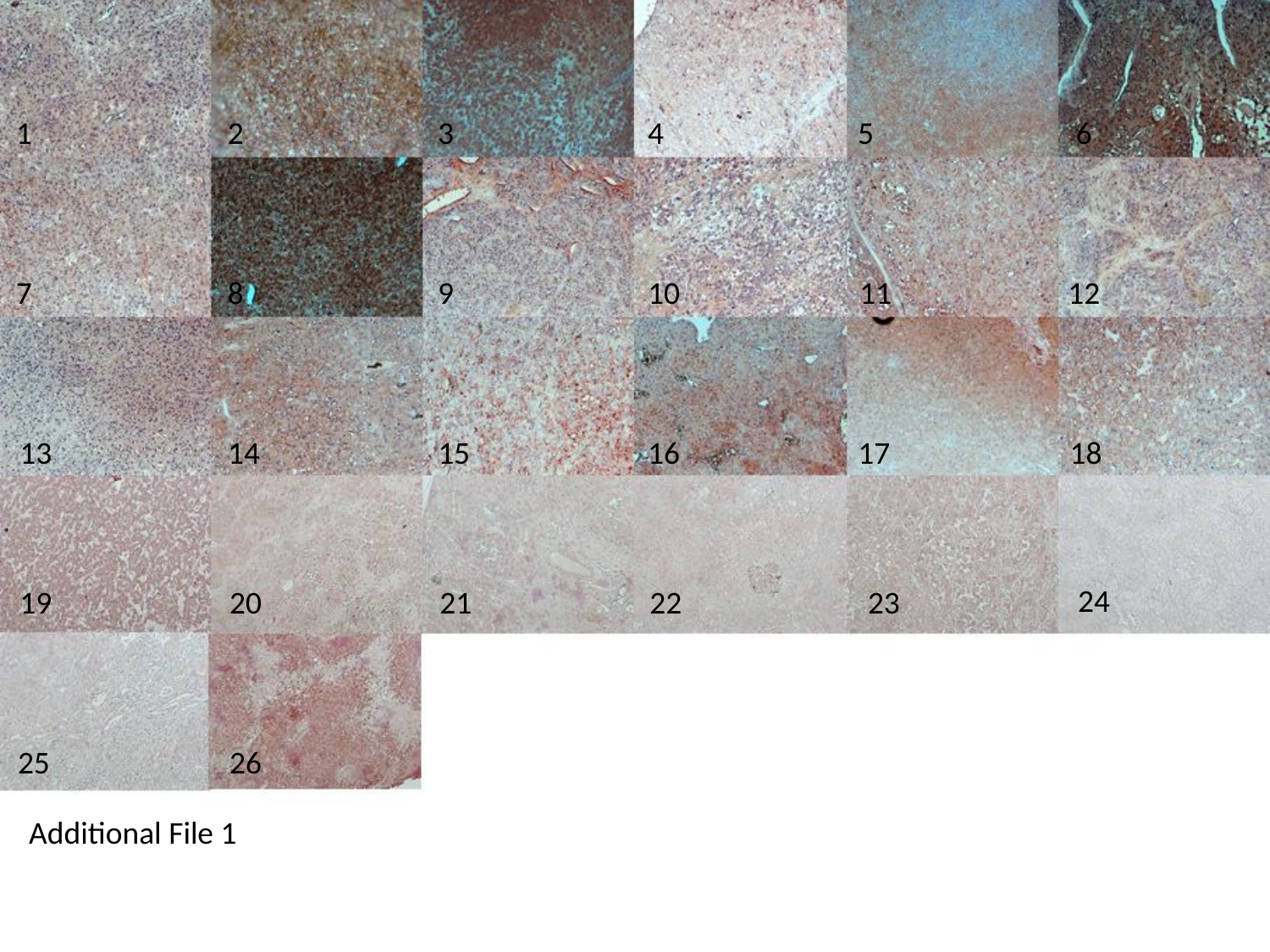

1
2
3
4
5
6
7
8
9
10
11
12
13
14
15
16
17
18
24
19
20
21
22
23
25
26
Additional File 1

Supplement: Supplementary file 1 — Additional file 1. Overview on the expression of FGFR2 in different adrenocortical carcinomas (n = 26). Images were captured using 100× magnification. [file 13104_2020_5110_MOESM1_ESM.pptx]

## Slide 1
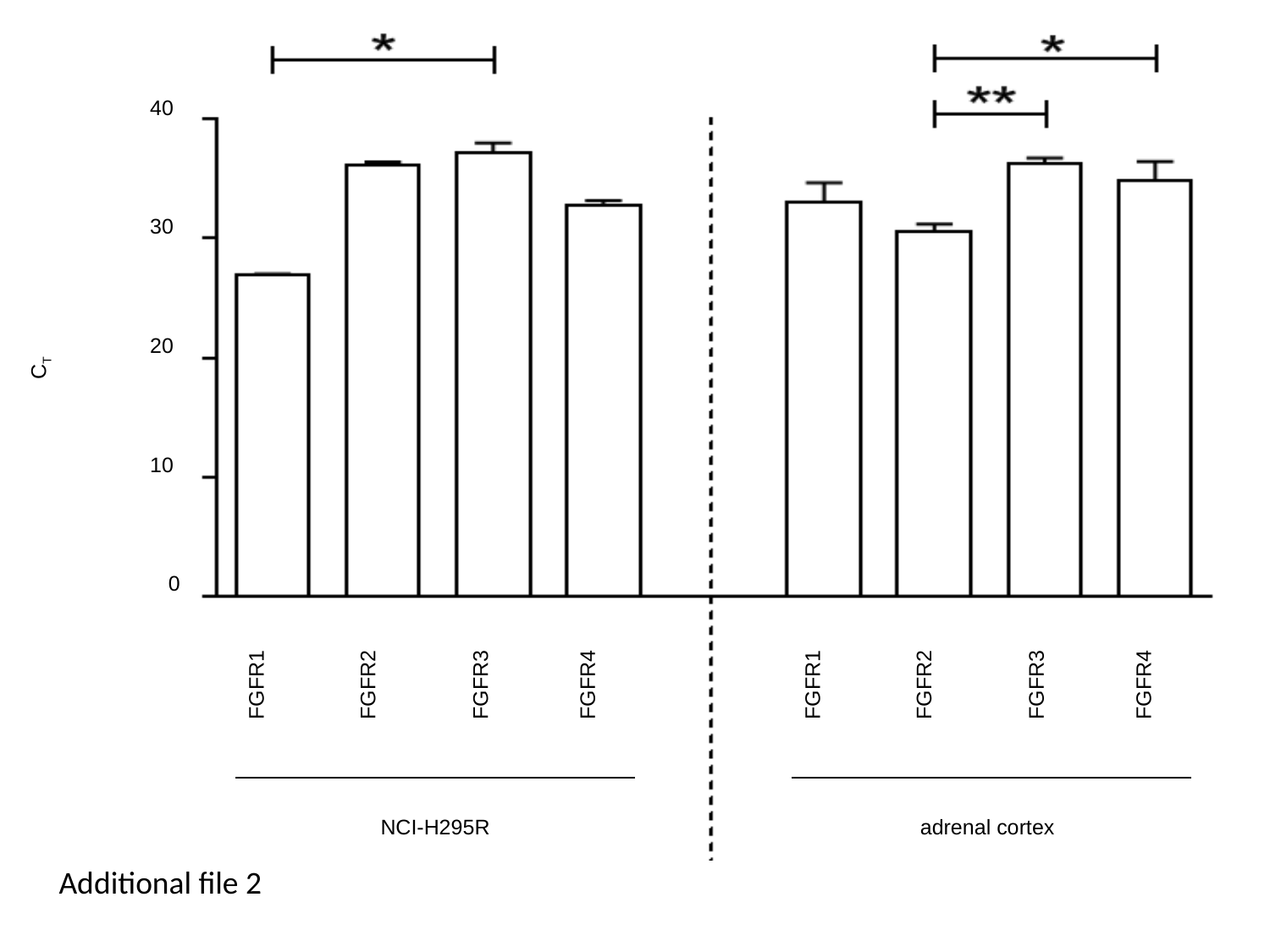

40
30
20
10
0
CT
FGFR1
FGFR2
FGFR3
FGFR4
FGFR1
FGFR2
FGFR3
FGFR4
adrenal cortex
NCI-H295R
Additional file 2

Supplement: Supplementary file 2 — Additional file 2. mRNA expression of FGFRs in the human adrenocortical cell line NCI-H295R and in normal human adrenal cortex. Asterisks indicate significant differences of the cycle thresholds (*P < 0.05, **P < 0.01). Due to the lack of references genes a valid quantitative analysis based on this data is not possible. [file 13104_2020_5110_MOESM2_ESM.pptx]
